# Supplementary material for: Asynchronous magnetic resonance elastography: Shear wave speed reconstruction using noise correlation of incoherent waves
Source: Magn Reson Med. 2022 Oct 27;89(3):990–1001. doi: 10.1002/mrm.29502 (PMC9792433; doi:10.1002/mrm.29502)
Supplement: Supplementary file 1 — DATA S1 MATLAB code used to generate the numerical simulations of Figure 1 [file MRM-89-990-s002.zip › k-Wave/helpfiles/writeMatrix.html]

writeMatrix :: Functions (k-Wave)


# writeMatrix

Write MATLAB matrix to a k-Wave HDF5 file.

## Syntax

```
writeMatrix(filename, matrix, matrix_name)
```

## Description

`writeMatrix` writes a MATLAB matrix to an existing HDF5 file. If the HDF5 file specified by filename doesn't exist, it is automatically created. Matrix attributes required by the k-Wave C++ code are automatically added. All variables are stored as 3D matrices (with the size of unused dimensions set to 1), and complex matrices are reorganised to C++ format before storing.

## Inputs

|  |  |
| --- | --- |
| `filename` | name of HDF5 file to write matrix to |
| `matrix` | data to write |
| `matrix_filename` | string containing the name of the matrix |

## See Also

`h5write`, `writeAttributes`, `writeFlags`, `writeGrid`
